# Alveolar Antral Artery: Review of Surgical Techniques Involving this Anatomic Structure

**Published:** 2014-04

**Authors:** Amin Rahpeyma, Saeedeh Khajehahmadi

**Affiliations:** 1 *Oral and Maxillofacial Diseases Research Center, School of Dentistry, Mashhad University of Medical Sciences, Mashhad, Iran.*; 2 *Dental Research Center, Schoolof Dentistry, Mashhad University of Medical Sciences, Mashhad, Iran.*

**Keywords:** Artery, Open sinus lift, Caldwell-Luc, Dental Implant

## Abstract

**Introduction:**

The horizontal bony canal in the lateral maxillary wall is the site of anastomosis between the arterial branches from the posterior superior alveolar artery (PSAa) and the infraorbital artery. This anatomic structure is known as the ‘alveolar antral artery’.

**Materials and Methods:**

We performed a literature review. The anatomic location of the alveolar antral artery in the lateral maxillary sinus wall was researched and its importance in surgical procedures routinely performed on this bony wall discussed.

**Results:**

This artery can be accidentally involved during surgical procedures on the lateral maxillary sinus wall, such as open sinus lift surgery, horizontal osteotomy of the maxilla, Le Fort I fracture treatment, and Caldwell-Luc surgeries.

**Conclusion:**

The alveolar antral artery is an important anatomic structure in the lateral maxillary sinus wall. A preoperative cone beam computed tomography (CBCT) scan can be used as a good diagnostic procedure to reduce surgical complications in suspected cases as well as conditions that may involve this artery.

## Introduction

**Fig 1:**
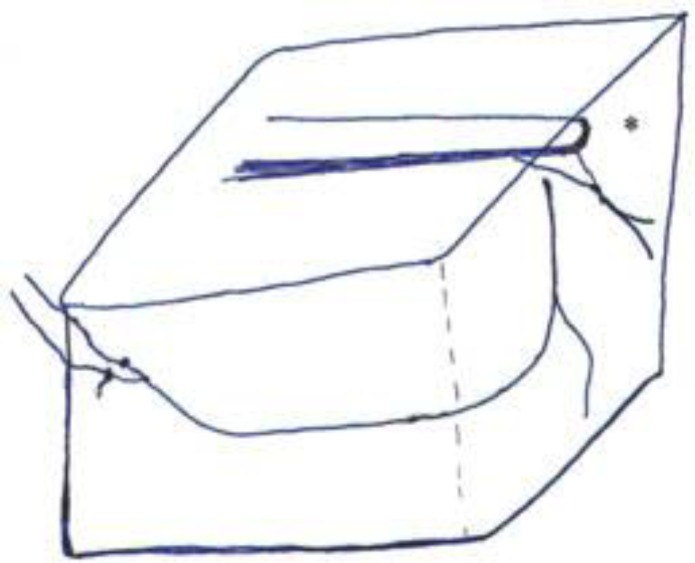
Schematic illustration showing alveolar antral artery as the anastomosing branch between posterior superior alveolar artery and infraorbital artery.*shows anterior superior alveolar artery

The horizontal bony canal in the lateral maxillary wall is a site of anastomosis between arterial branches from the posterior superior alveolar artery (PSAa) and the infraorbital artery (Fig.1) (1). 

From this artery, which is known as the alveolar antral artery, a number of vascular branches supply the maxillary sinus mucous membrane, periosteal tissue, and anterolateral wall of the maxillary sinus (2). Surgical procedures applied to this wall include open sinus lift, Caldwell-Luc surgery, and Le Fort I osteotomy, while osteosynthesis for the treatment of maxillary fractures may also involve this artery. Surgical manipulation of this artery during these procedures can lead to hemorrhage (3). 

In this review, examples of such complications along with solutions and routes to avoid this important anatomic structure are presented.

*Review of surgical techniques involving the anterolateral wall of the maxillary sinus*

Open sinus lift surgery

**Fig 2:**
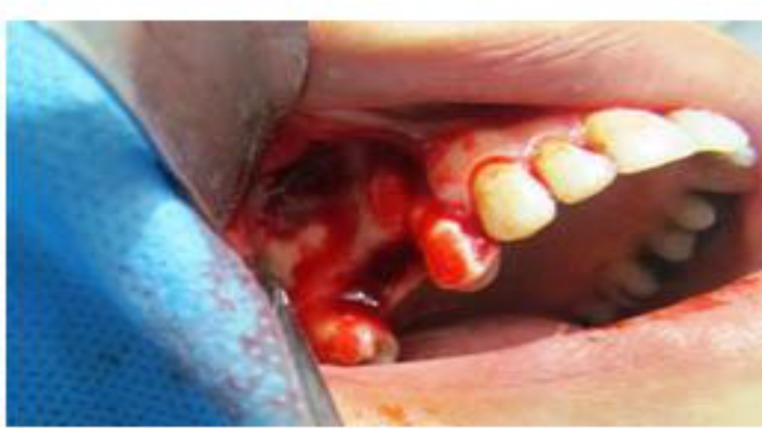
Bony window in open sinus lift surgery. Hemorrhage from alveolar antral artery can obscure vision.

Surgical involvement of the canal during bony window preparation can lead to hemorrhage. This bleeding will obscure the surgical field of vision and increase the chance of maxillary sinus membrane perforation. If the canal involved is large (diameter, >3 mm) there is also risk of severe bleeding (Fig. 2).

*Caldwell-Luc Surgery*

**Fig 3:**
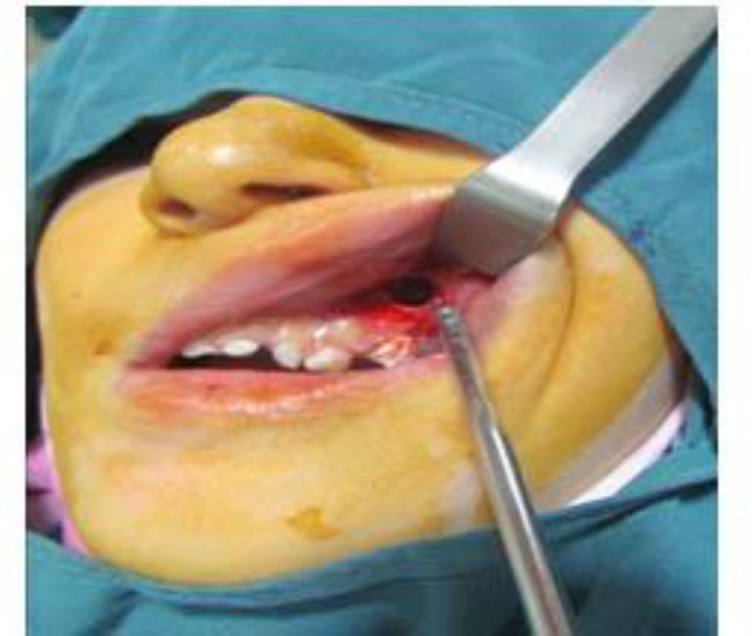
Access to the maxillary sinus via Caldwell-Luc operation

Confronting this canal and transacting it can lead to hemorrhage. Simultaneous bleeding from the disturbed intra-sinus lesion can further complicate the situation. The surgeon should be aware of the possible origin of bleeding from the periphery of the cut bony access (Fig. 3). Control of this bony canal hemorrhage could be performed using bone wax, electrocautery, or compression of the bone between the beaks of a hemostat.

*Le Fort I osteotomy*

**Fig 4:**
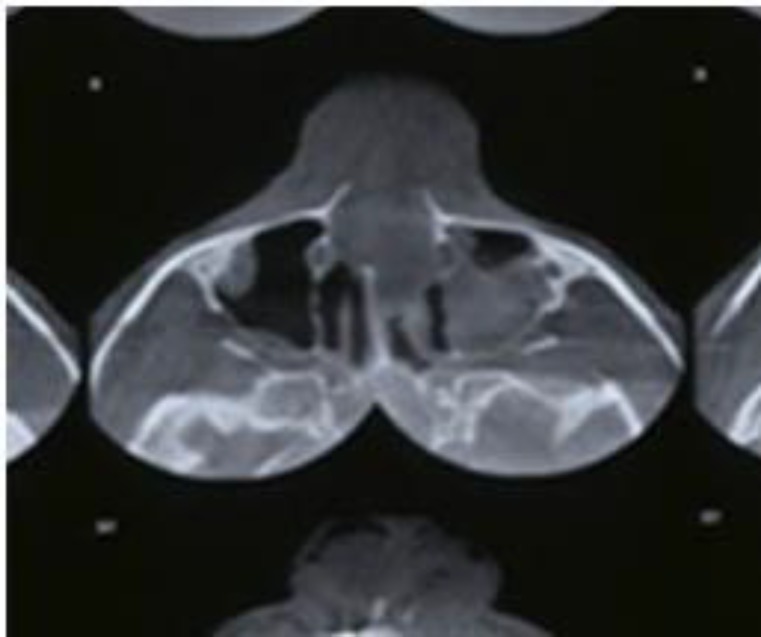
Postoperative CBCT of maxillary orthognathic patient with delayed nose bleeding, with origin at lateral maxillary wall

Osteotomy in the anterior maxillary bony wall can cause damage to the alveolar antral artery and result in hemorrhage. In some cases, the hemorrhage may not be controlled until complete maxillary down fracturing. This surgical procedure may last several minutes and result in massive blood loss requiring blood transfusion in elective surgery. Some delayed postoperative nasal bleeding in these patients can be attributed to this artery (Fig. 4).

*Maxillary fractures*

**Fig 5:**
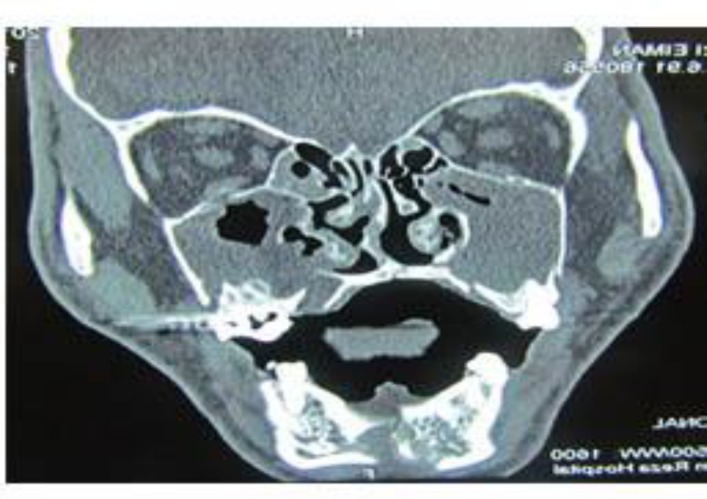
Opaque maxillary sinus due to fracture and severing of alveolar antral artery

A Le Fort I fracture involves bilateral fracture lines above the maxillary tooth roots. Sometimes this fracture line and displacement of the fractured bone can lead to hemorrhage from the alveolar antral artery that is manifested as nose bleeding not controllable with nasal packing (anterior and posterior). Another role for this artery in midface trauma patients (Le Fort and zygoma) is correlation between the clinical signs of nose bleeding and blood contaminated sputum. Severing this artery at the fracture line can lead to opaque maxillary sinus (Fig. 5). In contrast, clear maxillary sinus in the presence of a bony fracture in the anterior maxillary wall is almost always impossible. The final and most important note is the mistaken diagnosis of the alveolar antral artery as a fracture line. In this situation, confronting clinical symptoms and clean maxillary sinus cavity in obtained graphies is helpful.

## Materials and Methods

We performed a literature review. The anatomic location of the alveolar antral artery in the lateral maxillary sinus wall was researched and its importance in surgical procedures routinely performed on this bony wall discussed.

## Results

This artery can be accidentally involved during surgical procedures on the lateral maxillary sinus wall, such as open sinus lift surgery, horizontal osteotomy of the maxilla, Le Fort I fracture treatment, and Caldwell-Luc surgeries.

## Discussion

The alveolar antral artery is a constant artery in the lateral maxillary sinus wall. This wall is an osseous zone with a great density of vascular canals (4). It is an intraosseous anastomosis between the PSAa and infraorbital artery. Extraosseous anastomosis was reported by Traxler in a cadaveric study to be present in 44% of dissections (5). This extraosseous anastomosis is responsible for hematoma formation after high tuberosity nerve block injection (6). The alveolar antral artery was first described in the literature from 1937 (7). Studies on the prevalence of this artery by cadaveric and cone beam computed tomography (CBCT) scans have revealed different results. In cadaver studies there is a 100% prevalence rate of this anastomosis, while CBCT studies report the prevalence as 47–67% (8–11). This difference could be explained by the inability of CBCT to show arteries with a diameter less than 0.5 mm, as well as the fact that some alveolar antral arteries are subperiosteal to the schneiderian membrane and not localized in the bone.

**Fig 6:**
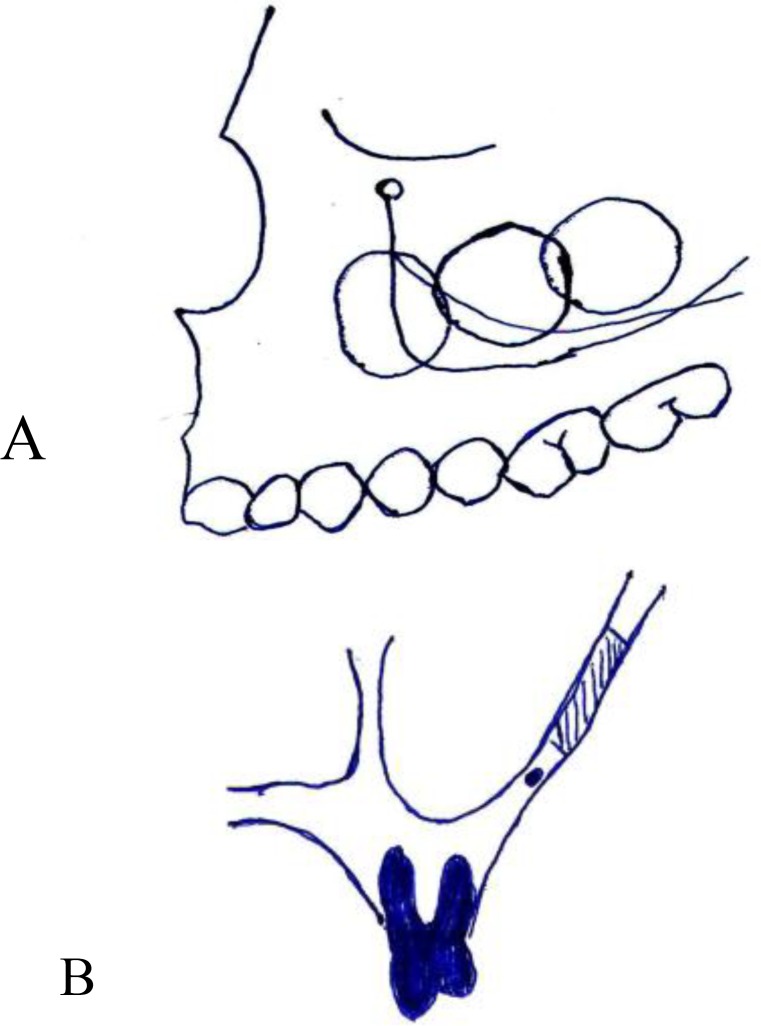
Changing ostectomy window design A: anterior/posterior direction B: superior location above the artery (schematic view).

Knowledge of this artery is essential for the surgeons who perform surgical procedures in this anatomic region. A preoperative CBCT alerts the surgeon to the presence of the alveolar antral artery, its location, and diameter. The surgeon is therefore able to make small modifications in the surgical procedure accordingly, such as changing the location of the osteotomy window in a Caldwell-Luc procedure and open sinus lift surgeries (Fig. 6) (12,13).

**Fig 7:**
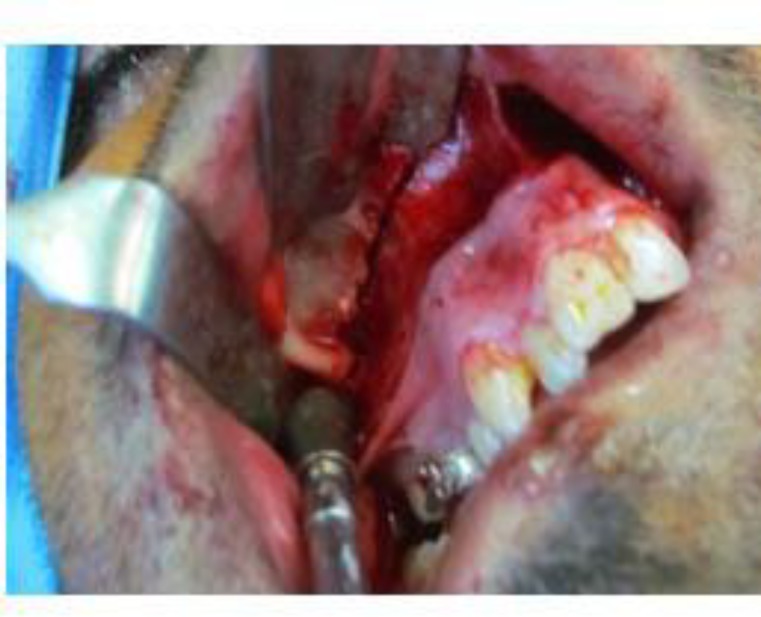
Stepped LeFort I osteotomy

Changing horizontal bony cuts in a maxillary Le Fort I osteotomy to a stepped design can reduce the possibility of surgical involvement of this artery in some cases (Fig.7). In maxillofacial trauma patients, attention to this artery can help the surgeon achieve a correct diagnosis and offer practical management plans, particularly in some cases of profuse nose bleeding after midface fractures, with the source of bleeding in the lateral maxillary wall (14). 

**Fig 8:**
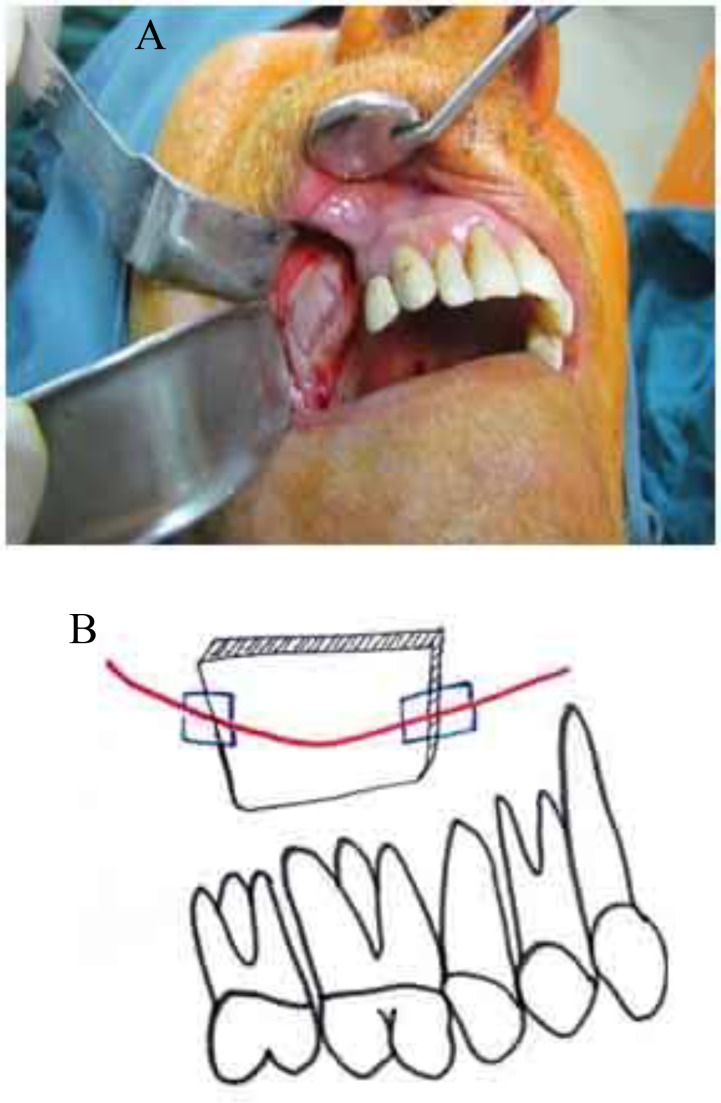
A) Final bone cuts are made near the alveolar antral artery in a condition when involving the artery is inevitable.

The same is true for Le Fort I orthognathic patients. Preserving this artery may have a beneficial role in preventing segmental ischemia in orthognathic and trauma patients. Trauma to this artery during maxillary osteosynthesis for fracture (Le Fort and zygoma) or orthognathic surgery is not important. Hemorrhage after drilling from bone holes will occur, but will immediately stop after screw tightening. If it is not possible to change the design of the osteotomy during open sinus lift surgery or zygomatic process donor-site preparation to obtain bone graft, the last bone cuts should be made near the alveolar antral artery and the surgeon and assistant should be ready to confront a bleeding episode (Fig. 8).

An additional consideration is the nerves that accompany this artery, and care must therefore be taken to inhibit injury to these intraosseous canals in order to prevent postoperative sensitivity disorders (15). Damage to these nerves during maxillary sinus antrostomy will not lead to upper teeth innervation injury, because the upper dental plexus is located within the thick maxillary alveolar process instead of the maxillary lateral sinus wall (16).

B) Schematic picture.

Attention to the vascular anastomosis in the lateral maxillary sinus wall is important for designing a vascularized pedicled bone flap for access to the maxillary sinus wall. This anastomosis, concomitant with overlying periosteal tissue or underlying maxillary sinus membrane, will guarantee the blood supply to the elevated vascular pedicled bone flap (17,18). 

Maintenance of such anastomosis is important to support bone graft neoangio- genesis in open sinus lift surgery, in which bone graft is inserted under elevated maxillary sinus membrane to increase the available alveolar bone for dental implant insertion (19).

## Conclusion

The alveolar antral artery is an important anatomic structure in the lateral maxillary sinus wall. It can interfere with surgical procedures for trauma, orthognathic,implant, and removal of pathologic lesions in the maxillary sinus. A preoperative CBCT scan can be used as a good diagnostic tool to reduce surgical complications in suspected cases as well as conditions that may involve this artery. However if the involvement of the alveolar antral artery during the surgical procedures is inevitable, or if it occurs accidentally, a number of solutions presented and described in this review could be very useful to control the situation.
